# Identity by Descent Mapping of Founder Mutations in Cancer Using High-Resolution Tumor SNP Data

**DOI:** 10.1371/journal.pone.0035897

**Published:** 2012-05-02

**Authors:** Eric Letouzé, Aliou Sow, Fabien Petel, Roberto Rosati, Bonald C. Figueiredo, Nelly Burnichon, Anne-Paule Gimenez-Roqueplo, Enzo Lalli, Aurélien de Reyniès

**Affiliations:** 1 Programme Cartes d'Identité des Tumeurs, Ligue Nationale Contre Le Cancer, Paris, France; 2 Instituto de Pesquisa Pelé Pequeno Principe and Faculdades Pequeno Principe, Curitiba PR, Brazil; 3 INSERM, UMR970, Paris Cardiovascular Research Center, Paris, France; 4 Assistance Publique-Hôpitaux de Paris, Hôpital Européen Georges Pompidou, Service de Génétique, Paris, France; 5 Université Paris Descartes, Sorbonne Paris Cité, Faculté de Médecine, Paris, France; 6 Institut de Pharmacologie Moléculaire et Cellulaire CNRS UMR 6097, and Université de Nice – Sophia Antipolis, Valbonne, France; Aarhus University, Denmark

## Abstract

Dense genotype data can be used to detect chromosome fragments inherited from a common ancestor in apparently unrelated individuals. A disease-causing mutation inherited from a common founder may thus be detected by searching for a common haplotype signature in a sample population of patients. We present here FounderTracker, a computational method for the genome-wide detection of founder mutations in cancer using dense tumor SNP profiles. Our method is based on two assumptions. First, the wild-type allele frequently undergoes loss of heterozygosity (LOH) in the tumors of germline mutation carriers. Second, the overlap between the ancestral chromosome fragments inherited from a common founder will define a minimal haplotype conserved in each patient carrying the founder mutation. Our approach thus relies on the detection of haplotypes with significant identity by descent (IBD) sharing within recurrent regions of LOH to highlight genomic loci likely to harbor a founder mutation. We validated this approach by analyzing two real cancer data sets in which we successfully identified founder mutations of well-characterized tumor suppressor genes. We then used simulated data to evaluate the ability of our method to detect IBD tracts as a function of their size and frequency. We show that FounderTracker can detect haplotypes of low prevalence with high power and specificity, significantly outperforming existing methods. FounderTracker is thus a powerful tool for discovering unknown founder mutations that may explain part of the “missing” heritability in cancer. This method is freely available and can be used online at the FounderTracker website.

## Introduction

With the advent of SNP arrays and next-generation sequencing, it is now possible to genotype millions of SNPs in a single experiment, at low cost. This has made it feasible to detect DNA regions inherited from a common ancestor in two apparently unrelated individuals. Two stretches of DNA are said identical by descent if they are identical due to common ancestry. The detection of identity by descent (IBD) in populations is considered a highly promising approach to the linkage mapping of disease genes. Indeed, linkage analyses are usually carried out in small pedigrees containing closely related individuals. Consequently, the IBD segments identified tend to be too long to delineate focal regions of interest with a restricted number of candidate genes. By contrast, tracts of IBD in unrelated individuals rarely span more than a few centimorgans (cM). Population-based linkage studies are thus very useful for the fine delineation of candidate loci [1]. Several powerful methods have been described for detecting tracts of IBD between pairs of individuals, based on dense phased [2], [3] or unphased [4], [5] genotyping data. These methods are useful for pairwise IBD detection, but population-based linkage mapping requires the detection of genomic regions with a significant excess of IBD in cases of disease with respect to controls. Two methods were recently proposed for the detection of IBD between multiple individuals: a Markov Chain Monte Carlo approach (MCMC) [6], and DASH (DASH Associates Shared Haplotypes) [7], a graph-based algorithm which builds upon pairwise IBD segments to infer clusters of IBD individuals. A major limitation of MCMC is that it involves intense computation and is therefore not suitable for the analysis of large data sets consisting of high-density SNP profiles. DASH is much faster, but only returns a list of haplotypes that are identical by descent in several samples. Significant enrichment in disease cases may then be assessed through a statistical association test. However, this approach only takes into account the frequency of conserved haplotypes, and not their length. Yet, a long haplotype, even encountered in a few cases, is likely to indicate the presence of a founder mutation.

Our method is specifically dedicated to the mapping of cancer genes. One key feature of cancers is that tumor cells accumulate chromosome aberrations providing a growth advantage during cancer progression, such that the tumor genome eventually becomes a highly rearranged version of the constitutive genome of the patient. Besides, the location of somatic alterations is partly driven by the presence of risk alleles in the germline genome [8]. In particular, patients inheriting a germline mutation in a tumor suppressor gene frequently lose its normal counterpart in cancer cells, revealing the mutant phenotype, as in Knudson's two-hit model [9]. Thus, germline mutations are often located in regions of loss of heterozygosity (LOH). This feature is particularly useful for the linkage mapping of cancer genes. Firstly, regions of LOH can be used to prioritize the search for recurrent IBD. Secondly, when a genomic region undergoes LOH, only one of the two parental chromosomes remains in the tumor DNA, so the haplotype of this chromosome is given directly by the tumor SNP profile, without the need for a haplotype-phasing step. Minimal regions of LOH are thus an ideal starting point for the detection of recurrent IBD in a population sample of tumors.

**Figure 1:**
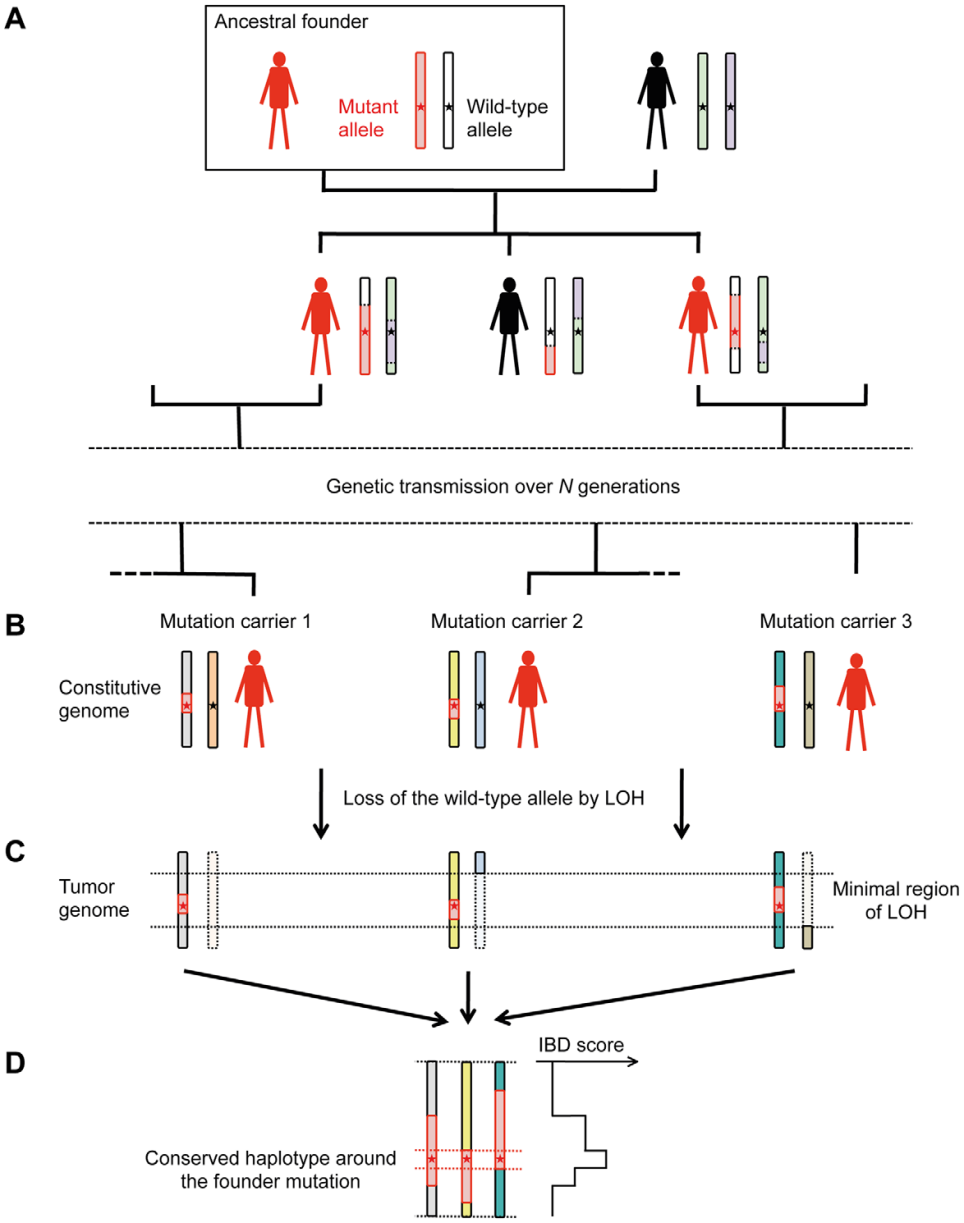
IBD mapping of founder mutations in recurrent regions of LOH. This diagram illustrates the principles underlying our method. A founder mutation (red star on the schematic diagram of chromosomes) spreads through a population within a chromosome fragment (in red) inherited from the ancestral founder (A). Due to crossing-overs (dashed lines) between homologous chromosomes at meiosis, this chromosome fragment is shortened over generations, such that mutation carriers (indicated in red) eventually harbor only a short identical by descent (IBD) haplotype around the mutant gene (B). In addition, the wild-type counterpart of germline mutations is frequently lost by LOH in tumors, such that the founder mutation typically lies within a minimal region of LOH (C). As a result, the founder mutation is located within a haplotype conserved in each mutation carrier (peak IBD score), in the minimal region of LOH (D).

Our method is designed for detecting cancer-associated mutations inherited from a common founder, using recurrent IBD in minimal regions of LOH (Figure 1). Building upon the GERMLINE algorithm [3] for pairwise IBD detection in phased haplotypes, we describe a scoring approach that takes into account both the frequency and length of IBD segments for the characterization of significantly conserved haplotypes in a set of tumor samples. We validate this approach on two real cancer data sets. In the first dataset, comprising 13 childhood adrenocortical tumors from southern Brazil, we delineate a conserved haplotype spanning 520 kb around the previously reported p.R337H *TP53* founder mutation. In the second dataset, comprising 30 pheochromocytomas and paragangliomas, we detect a novel founder mutation of the *SDHD* gene, present in only two samples. Finally, we show with simulated data that FounderTracker detects conserved haplotypes of low prevalence with high power and specificity, significantly outperforming existing methods.

## Results

### Detecting conserved haplotypes in recurrent regions of LOH

Our strategy for the population-based linkage mapping of founder mutations in cancer is illustrated in Figure 1. A mutation spreading through a population is transmitted within a chromosome fragment from the common ancestor, which becomes smaller from generation to generation due to genetic recombination at meiosis. All mutation carriers are thus identical by descent to their common ancestor for the chromosome region harboring the germline mutation. In addition, the wild-type counterpart of the mutant gene is frequently lost by LOH in tumors occurring in germline mutation carriers. Our approach to the mapping of founder mutations using SNP array data thus involves (i) identifying recurrent regions of LOH, (ii) reconstructing tumor haplotypes in these regions, and (iii) searching for recurrent IBD in tumor haplotypes.

With one probe targeting each of the two alleles of each SNP, SNP arrays are an excellent tool for the genome-wide detection of LOH, and several algorithms have been described for this purpose [10], [11]. In this study, we use the Genome Alteration Print method [12] to detect LOH, defining a set of recurrent regions of LOH on the basis of their frequency in the tumor data set.

**Figure 2:**
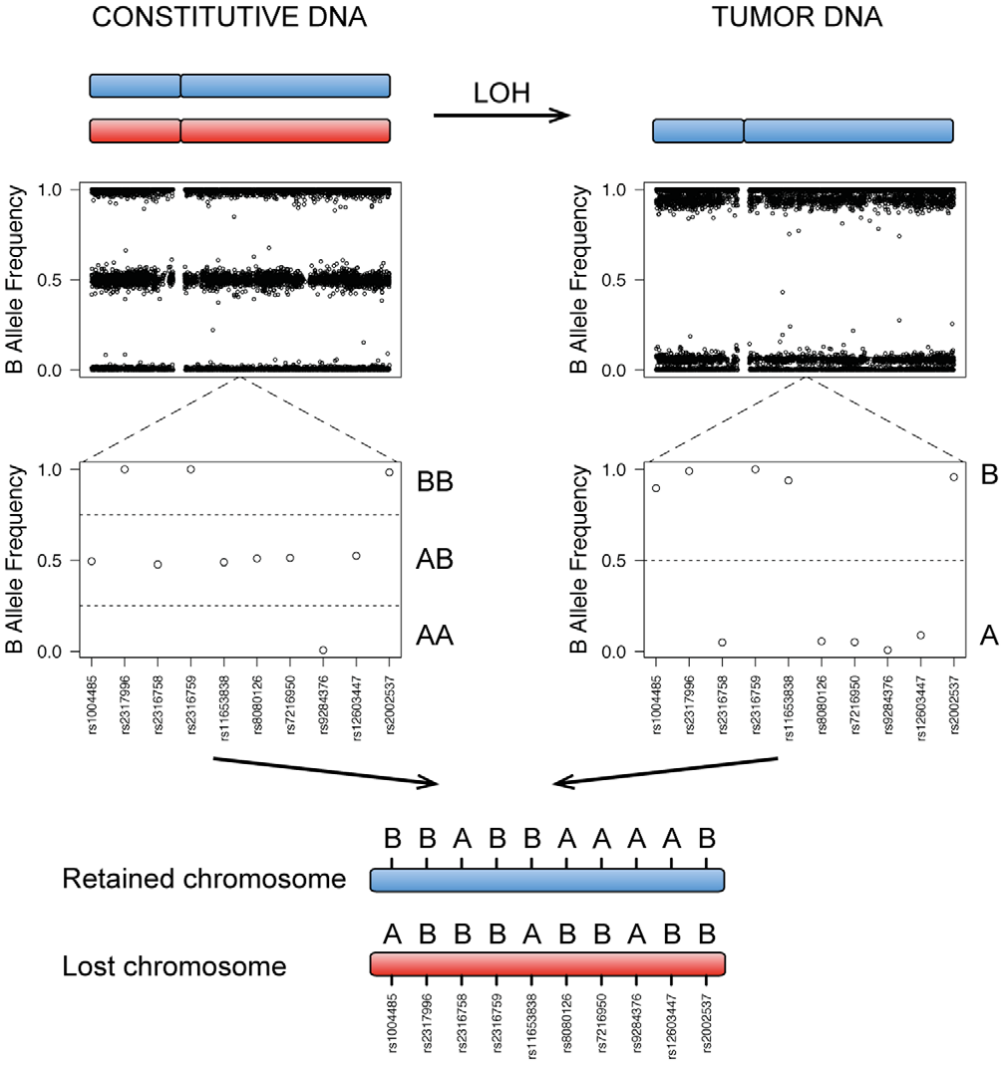
Haplotype inference from tumor SNP profiles. Genotypes can be inferred from SNP array data, with the B allele frequency (BAF), which characterizes the fluorescence ratio between the A and B alleles at each locus: *BAF = B/*(*A+B*). In constitutive DNA, each SNP is present as two alleles. The genotype of each SNP (AA, AB, or BB) can thus be determined from the BAF (0, 0.5 or 1, respectively), but not the haplotype of each chromosome. By contrast, if one of the two copies has been lost by LOH in the tumor, the tumor SNP profile directly reflects the haplotype of the chromosome that is retained in the tumor. If paired constitutive and tumor DNA samples are available, the haplotype of the lost chromosome can be reconstructed by comparing the two profiles.

We then reconstruct the haplotype of the retained allele in each tumor (Figure 2). As only one of the two chromosomes remains in the tumor due to LOH, the genotypes obtained for tumor DNA directly provide the haplotype of the chromosome retained in tumor cells. Incidentally, if constitutive DNA is also available, a comparison of the unphased genotypes of constitutive DNA with the tumor haplotype can be carried out to infer the haplotype of the lost chromosome. To estimate the error rate associated with this approach, we compared the haplotypes reconstructed for two tumors from the same patient in which the same chromosome was lost by LOH [13], and we obtained a very low error rate (<5×10^−5^).

**Figure 3:**
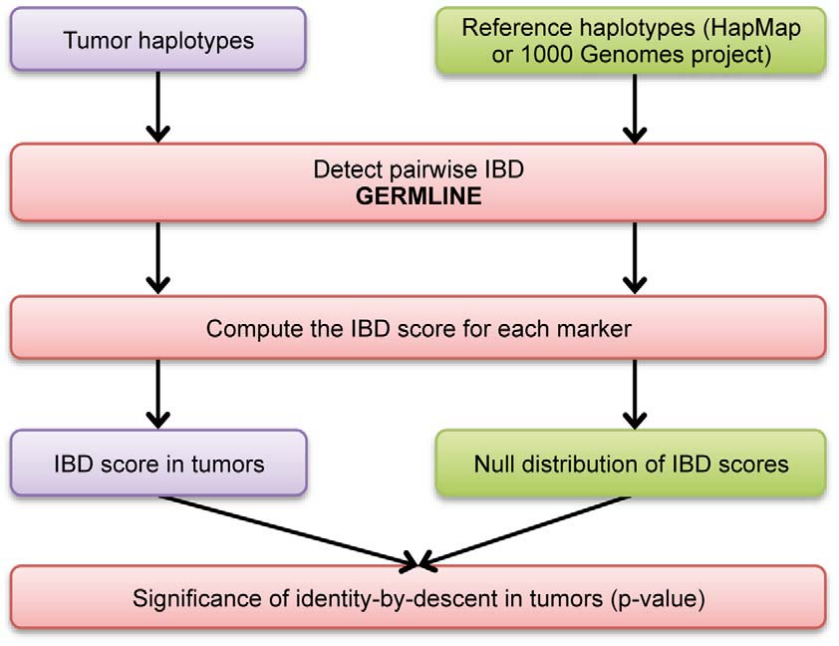
Detecting significantly recurrent IBD in a set of tumor haplotypes. The FounderTracker pipeline for recurrent IBD detection is divided into two steps. An IBD score is first calculated for each SNP marker in the tumor set of haplotypes. The IBD score is derived from the pairwise IBD segments identified with the GERMLINE algorithm. It takes into account their length and the allelic frequencies of SNPs within each segment. The IBD score for tumors is then compared with a null distribution established from a reference data set, to identify the SNPs with IBD scores significantly higher than expected by chance.

Finally, we search for significantly recurrent IBD in the set of reconstructed tumor haplotypes. The analytical pipeline for this step is represented in Figure 3. Segments of pairwise IBD are first identified with the GERMLINE algorithm [3]. We then assign a score to each pairwise IBD segment, taking into account both the number and the allelic frequencies of SNPs within each segment (see Materials and Methods). An IBD score is then calculated for each SNP marker, by summing the scores of all the pairwise IBD segments containing this SNP. Linkage disequilibrium (LD) must also be taken into account, because genomic regions of strong LD will systematically have high IBD scores (Figure S1). We therefore compare, for each SNP, the IBD score obtained for tumors with a null distribution of IBD scores established using a reference set of haplotypes from healthy controls (e.g. phased haplotypes from the 1,000 Genomes projects).

### Application to the case of childhood adrenocortical tumors in southern Brazil

**Figure 4:**
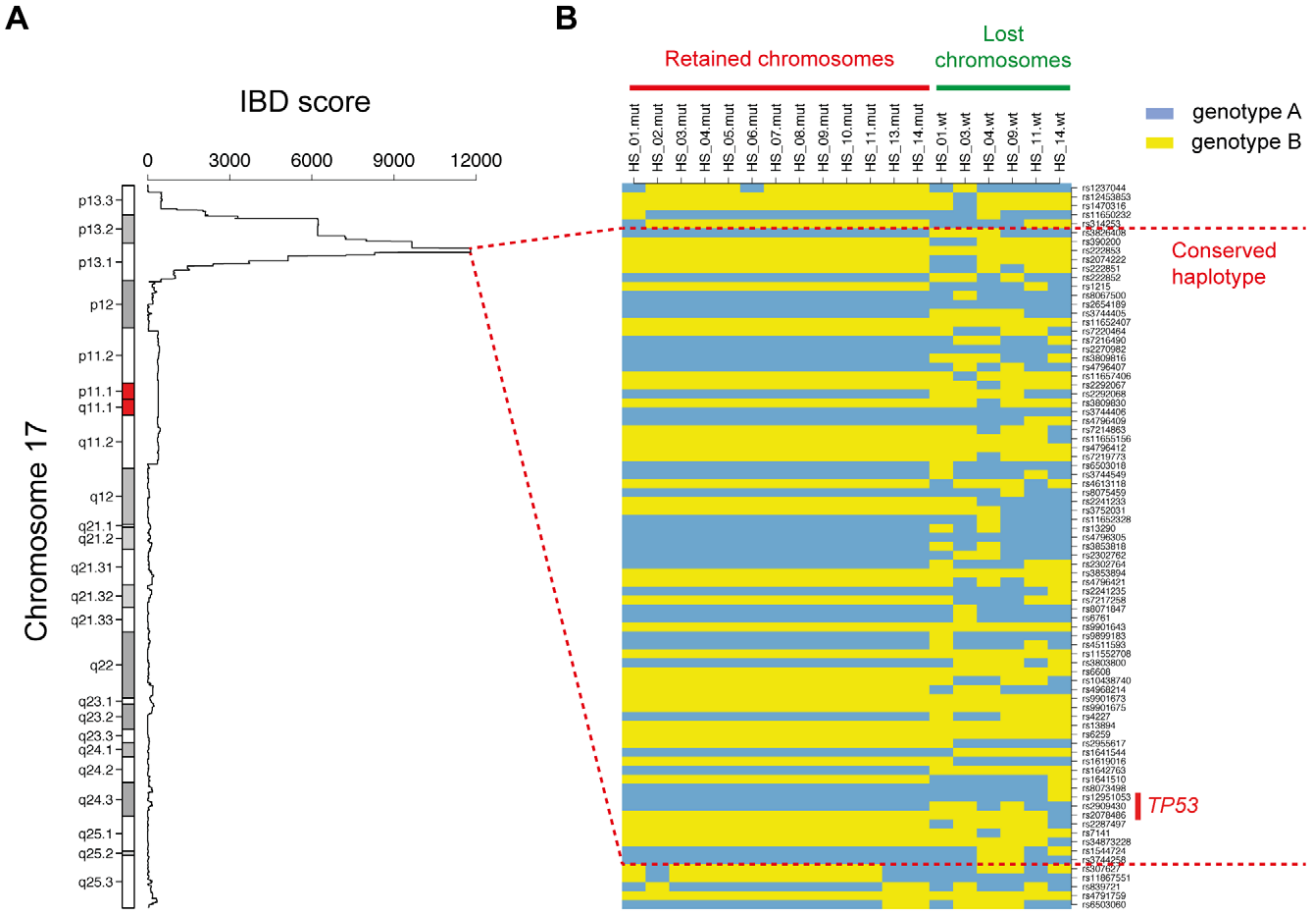
A conserved haplotype around the *TP53* founder mutation in childhood adrenocortical tumors. The IBD score calculated along chromosome 17 for the set of 13 childhood adrenocortical tumors (ACT) displays a significant peak at 17p13.1 (A). The sharp increases in the IBD score curve correspond to the boundaries of IBD segments in each pair of tumors. The peak region is represented in detail in panel B, with the haplotypes of the 13 chromosome copies retained in the tumors (harboring the mutant *TP53* allele), and the haplotypes of the chromosome copies lost by LOH (harboring a wild-type *TP53* allele) reconstructed for the six patients with matched normal blood samples. The haplotypes of wild-type alleles are all different, demonstrating the existence of different haplotypes for this genomic region in the population analyzed. By contrast, a haplotype common to all mutant alleles spans 470 kb upstream and 32 kb downstream from *TP53* (represented by a thick red line). This conserved haplotype corresponds to the chromosome fragment harboring the R337H *TP53* mutation inherited from the ancestral founder.

As a proof-of-concept, we first applied our methodology to the example of childhood adrenocortical tumors (ACT). These rare cancers (0.3–0.4 cases per year per million children under the age of 15 years) are exceptionally prevalent in southern Brazil (3.4–4.2 cases per million) [14], where they are almost invariably linked to a specific germline mutation of *TP53* (c.1010G>A, p.R337H) [15], [16], identified as a founder mutation [17]. We analyzed 13 Brazilian cases of ACT and six matched blood samples on Illumina 370K SNP arrays. All tumors were found to carry the p.R337H *TP53* mutation. We used the Genome Alteration Print method to detect LOH. This method identified two regions displaying LOH in all samples: a 4.4 Mb region on 11p15, and the entire chromosome 17. We then used FounderTracker to detect significantly recurrent IBD in these two regions. No significant region of IBD was detected in the 11p15 region (Figure S1), but highly significant IBD scores were obtained for the 17p13 region (q-value <2.2×10^–16^). In particular, the peak IBD score defined a 520 kb region around the *TP53* gene (Figure 4a). Detailed haplotype analysis revealed that the mutated copies retained in the 13 tumors displayed an identical haplotype for this region, whereas the wild-type copies reconstructed for the six patients with matched blood samples displayed different haplotypes (Figure 4b). This recurrent IBD segment thus corresponds to the chromosome fragment carrying the p.R337H *TP53* mutation inherited by all the patients from the common founder, which has not been disrupted by crossover in the lineage.

The mean length of pairwise IBD tracts around *TP53* was 5.4 cM (ranging from 0.88 cM to 19 cM, Figure S2). As pairwise IBD segments are estimated to have a length of *1/*(*2n*) Morgans after *n* generations (hence *2n* meiosis) [2], this suggests that the p.R337H *TP53* mutation occurred around nine generations ago.

### Application to the case of pheochromocytomas and paragangliomas

**Figure 5:**
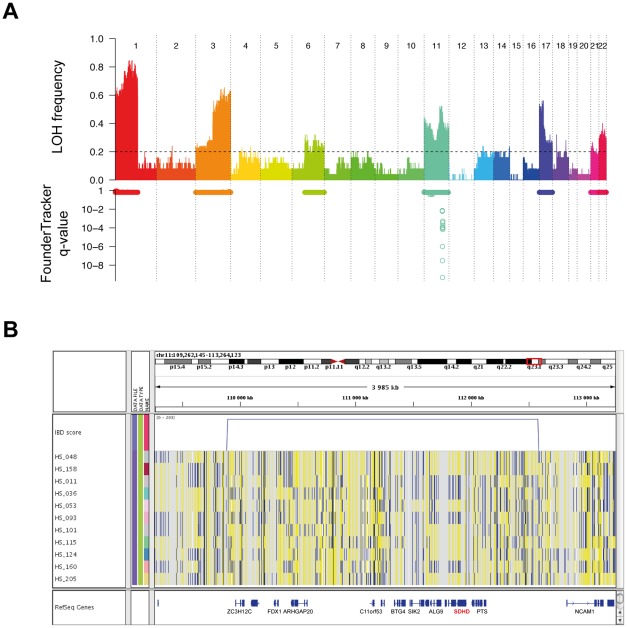
Detection of a long conserved haplotype around *SDHD* gene in two paragangliomas. (A) LOH analysis revealed 10 chromosome arms with LOH frequency >20% in our set of 30 pheochromocytomas and paragangliomas (top). These regions were analyzed using FounderTracker to detect conserved haplotypes, revealing a single significant region on chromosome arm 11q (bottom). (B) Visualization of the significant region identified on chromosome 11 with the Integrative Genomics Viewer [44]. The IBD score is represented as a blue line above tumor haplotypes. Haplotypes are represented as series of blue and yellow vertical lines, corresponding to SNPs with respectively “A” and “B” genotype, according to Illumina nomenclature. The significant region detected by FounderTracker corresponds to the long haplotype that is identical in tumors HS_048 and HS_158, and results in a high IBD score for this segment. This region contains 32 genes, including *SDHD* (indicated in red).

Pheochromocytomas and paragangliomas are neuroendocrine tumors arising from the adrenal medulla and from sympathetic or parasympathetic paraganglia tissues respectively. These tumors occur in the context of inherited cancer syndromes in ∼30% of cases [18]. Germline mutations associated with familial pheochromocytoma include mutations of *RET*, *NF1*, *VHL*, *TMEM127*, *MAX*, and genes encoding proteins from the succinate dehydrogenase complex (*SDHA*, *SDHB*, *SDHC*, *SDHD* and *SDHAF2*). Here, we applied our methodology for founder mutation discovery to a set of 30 pheochromocytomas/paragangliomas from unrelated patients. Samples were fully characterized in terms of germline mutations, and analyzed on Illumina 610K SNP arrays. LOH analysis revealed ten regions with LOH frequency above 20%, at 1p, 3p, 3q, 6q, 11p, 11q, 17p, 17q, 21q, and 22q (Figure 5A, top). For these regions, tumor haplotypes were reconstructed from the B Allele Frequency profiles, and analyzed with FounderTracker to detect conserved chromosome segments. This analysis revealed a 2.34 cM region with significant IBD at 11q23.1 (Figure 5A, bottom). Interestingly, this chromosome segment contains 32 genes including *SDHD*, and is identical by descent in two samples (HS_048 and HS_158, Figure 5B), which are precisely the two samples in our cohort for which a c.64C>T (p.Arg22X) germline *SDHD* mutation was identified (Table S1). This finding demonstrates that the p.Arg22X *SDHD* mutations in these two seemingly unrelated patients originate from a single founder, and further validate the relevance of our method for detecting founder mutations in cancer.

### Evaluation of the power of the method with simulated data

Childhood ACT is an ideal case study for population-based linkage mapping. As this disease is extremely rare in absence of the p.R337H *TP53* founder mutation, all of the samples selected through a random sampling of tumors bear this mutation. In most contexts, tumors attributable to a given founder mutation account for only a subset of cancers in a population. We have shown with the pheochromocytoma/paraganglioma data set that FounderTracker was able to detect a founder mutation present in only 2 out of 30 samples (7%). To further evaluate the performance of our method, we generated simulated data into which we artificially introduced conserved haplotypes of different lengths and frequencies. We benchmarked FounderTracker by analyzing the same simulated data with the DASH algorithm, recently developed to detect clusters of individuals sharing an identical haplotype [7], which we adapted to our purposes (see Material and Methods). This approach gave good results with the childhood ACT data set (Figure S3), validating its relevance as a benchmark method.

**Figure 6:**
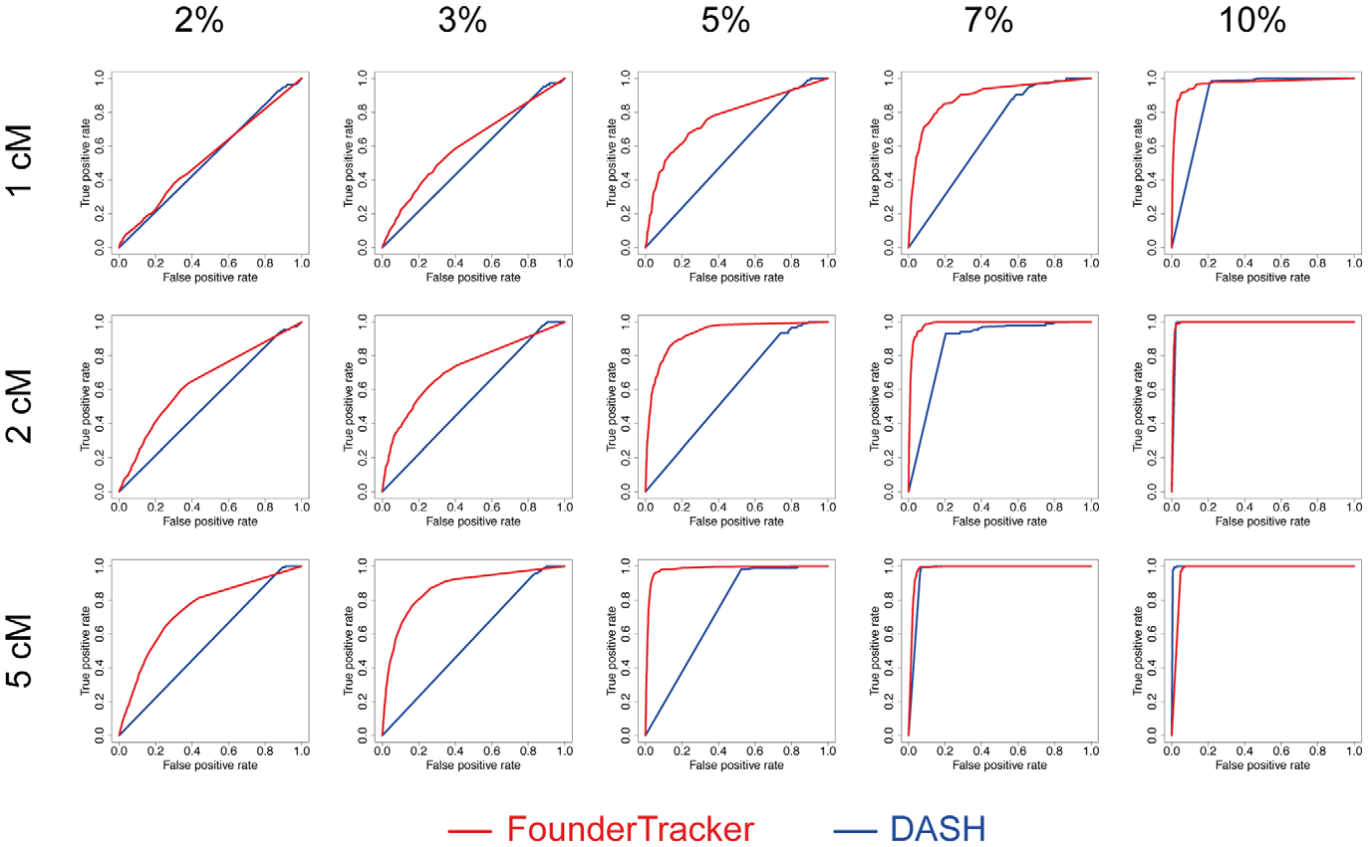
Power analysis with simulated data. The performance of FounderTracker was assessed under various conditions, by comparison with the DASH method. The ability of each method to detect conserved haplotypes was established as a function of haplotype length (1 to 5 cM) and prevalence (2 to 10% of the samples). For each condition, the mean ROC curve was established by applying each method to 100 simulated datasets.

All haplotypes conserved in ≥50% samples were successfully detected by either method (Table 1). Both FounderTracker and DASH were able to detect haplotypes conserved in ≥15% samples with good power (>0.9), but FounderTracker significantly outperformed DASH in the low frequency range (≤10%) (Table 1, Figure 6). The ROC curves indicate that FounderTracker detects recurrent IBD segments with high sensitivity and specificity down to a minimum frequency that depends on the length of IBD segments. IBD tracts of 5 cM are detected with good power (>0.9) from a frequency of 5%, whereas short segments of 1 cM are detected with the same power only at frequencies ≥15%. The false discovery rate, calculated across all simulations, was negligible for both methods (respectively 8.6×10^−3^ and 5.4×10^−4^ for FounderTracker, and DASH).

**Table 1:** Power to detect conserved haplotypes as a function of haplotype length and prevalence.

Size	Method	2%	3%	5%	7%	10%	15%	20%	50%
**1 cM**	FounderTracker	0.02	0.02	0.11	0.29	0.65	0.94	0.9	1
	DASH	0	0	0	0.03	0.36	0.9	0.95	1
**2 cM**	FounderTracker	0.01	0.04	0.35	0.83	0.98	0.99	1	1
	DASH	0	0	0	0.13	0.8	1	1	1
**5 cM**	FounderTracker	0.06	0.27	0.92	1	1	1	1	1
	DASH	0	0	0	0.47	1	1	1	1

The simulated data presented here correspond to the SNP content of the Illumina 1M-Duov3 beadchip (1,199,187 markers). To assess the impact of SNP density on the performance of the method, we analyzed the same simulated data, restricted to the sole 373,397 SNPs of the Illumina HumanCNV370-Quad chip. Although there was a slight degradation in the detection of short haplotypes (0.5 cM) with Illumina CNV370 array, we obtained a comparable level of performance with both marker densities (Figure S4). It has been estimated that 85% of the human genome is spanned by haplotype blocks of 10 kb or larger in European samples [19] so that the majority of common SNPs are well captured by whole-genome genotyping arrays containing 100,000 markers or more, either directly or through linkage disequilibrium [20]. Our results are consistent with this figure and provide important information about the range of applicability of our method, suggesting that the power achievable with the current generation of arrays is close to the maximal power of IBD mapping.

The running time of FounderTracker is linearly associated with the number of samples and SNP markers analyzed. For example, the processing of the childhood ACT dataset (13 samples, 9,762 SNPs) took 4 min 07 seconds, and the longest run in our simulations, for a dataset of 100 chromosome 1 haplotypes (80,158 SNPs), took 2 hours 48 minutes on a single core of an Intel Xeon X5470 Quad-Core processor running at 3.33 GHz. However, as noted above, little increase in power would be expected from the analysis of denser SNP data, so the amount of data analyzed can be constrained, in terms of markers, to the current SNP content of high-definition arrays.

## Discussion

Most of the founder effects detected to date were revealed by targeted studies of polymorphic markers around well known cancer-related mutations. We introduce here the first systematic approach for detecting founder mutations on a genome-wide basis, from dense SNP profiles of tumor samples.

SNP arrays are widely used in cancer research for two main purposes: the discovery of germline risk variants in linkage or association studies, and the identification of recurrent chromosome rearrangements. Researchers typically study exclusively the germline genome for association studies, or the tumor genome for the analysis of chromosome rearrangements, but a combined analysis of risk alleles and chromosome aberrations has been shown to be fruitful [21]. In this study, we used LOH to prioritize regions of interest for IBD mapping. One of the limitations of this approach is that other mechanisms, such as mutation or promoter hypermethylation, may inactivate the wild-type counterpart of a mutant gene in the absence of LOH. However, previous studies have shown LOH to be a common “second hit” in germline mutation carriers [22], [23]. Our approach is thus likely to be relevant in a large proportion of cases. In addition, high-confidence haplotypes can be inferred from tumor SNP profiles in regions of LOH. IBD detection can thus be carried out with stringent parameters, making the method highly specific. Finally, LOH at a cancer gene locus is likely to be more frequent in mutation carriers than in non-carriers, as already shown for the *BRCA* genes [24]. By considering only samples with a LOH for each candidate region, we may thus be able to detect conserved low-frequency haplotypes that would not have been detected as significant by considering the whole tumor set.

No existing approach was exactly designed for the systematic detection of founder mutations in cancer, but the DASH method for detecting clusters of haplotypes sharing identity-by-descent could be easily adapted to our purposes. The difference between FounderTracker and DASH lies in the characterization of significantly recurrent IBD from pairwise IBD matches. DASH identifies clusters of haplotypes conserved in several samples. One can then test whether one of these haplotypes is significantly overrepresented in tumors, as compared to normal controls. A limitation of this approach is that, once clusters are identified, the association test does not take into account the length of conserved haplotypes. By contrast, the FounderTracker approach evaluates an IBD score that takes into account both the frequency and the length of conserved haplotypes. As a result, FounderTracker significantly outperformed DASH for detecting haplotypes conserved in ≤10% samples. This makes an important difference in terms of applications, since founder mutations are likely to be relatively rare in most cases. For instance, a founder mutation present in only 5% of tumors, lying within a conserved haplotype of mean length 5 cM, (like the p.R337H *TP53* mutation in Brazilian ACT) will be detected with a power of 0.92 with FounderTracker versus 0 with DASH (Table 1). Besides, the haplotype around *SDHD*, conserved in two paragangliomas, is not detected by DASH (Figure S5). Another existing method for detecting IBD in multiple individuals is the Markov Chain Monte Carlo (MCMC) approach [6]. However, this method is highly computation-intensive and the authors described its application to small data sets only (maximum of 15 samples and 1278 SNPs). When we tried to apply this approach to our set of data for childhood adrenocortical tumors, the method failed to converge after several days. By contrast, FounderTracker was able to process the same dataset in a few minutes. We conclude that MCMC is not very suitable for the analysis of high-definition SNP array data.

We show, with simulated data, that our method can detect conserved haplotypes of at least 0.5 cM in length (Figure S4). Chapman *et al.* showed that, after 100 generations of random mating in a growing population, IBD tracts would have a mean length of 0.6 cM [25]. Our method is therefore suitable for the detection of cancer-related mutations that appeared less than 100 generations ago. Since FounderTracker relies on the null distribution of IBD scores to identify significantly conserved haplotypes, we strongly encourage FounderTracker users to use reference samples of the same ancestry as the tumor samples to calculate null distributions. In absence of a Brazilian reference data set, we used the CEU data of the 1,000 Genomes project as a reference for the analysis of adrenocortical tumors, because the population of the Parana state is predominantly of European origin [26].

In terms of frequency, FounderTracker can detect, with high power, haplotypes conserved in more than 5% of samples, depending on the length of the IBD segments. The proportion of tumors harboring a founder mutation in a random population sample depends principally on the genetic diversity of the population and the prevalence of the disease. Rare cancers, such as childhood ACT, with a higher prevalence in a specific geographic area are ideal for IBD mapping, because almost all cases are due to the founder mutation. In common diseases, such as breast cancer, high-frequency founder mutations were initially described in geographically or culturally isolated populations. For example, the *BRCA1* and *BRCA2* founder mutations were initially discovered in the Ashkenazi Jewish population [27], [28] and in Finland [29], respectively. However, the extensive analysis of these two genes in numerous geographic areas has since revealed the existence of more than 20 different founder mutations in different regions [30]. These findings suggest that founder mutations may be more common than currently thought. FounderTracker can be used online through a web application, and the source code can be downloaded from our web page (http://cit2.ligue-cancer.net/FounderTracker).

## Materials and Methods

### Patients and ethics statement

Thirteen ACT patients from a Brazilian cohort [31] were included in this study. Ethical approval for the study was obtained from the Pequeno Príncipe Hospital Ethics Committee (Curitiba, Brazil) and CONEP (Federal Ethics Committee in Brasilia, Brazil) in 2009. In all cases, one of the parents or legal representatives signed an informed consent form approved by the local ethics committee. DNA was extracted from the tumor and peripheral blood as previously described [31]. *TP53* mutation analysis was performed as previously described [32].

Tumor samples from thirty patients with pheochromocytoma or paraganglioma from the French COMETE network, collected by the Georges Pompidou European Hospital, were included in this study. Ethical approval for the study was obtained from the institutional review board (CPP Paris-Cochin, January 2007). All patients provided written informed consent for the collection of samples and subsequent analyses. Tumor DNA was extracted as previously described [33]. Clinical *NF1* diagnosis and genetic testing for *RET*, *SDHA*, *SDHB*, *SDHC*, *SDHD* and *VHL* was performed as previously described [33].

### SNP array hybridization and preprocessing

The 13 adrenocortical tumor samples were analyzed with Illumina HumanCNV370-Duo v1.0 chips, containing 370,404 probes, and the six matched normal samples were analyzed with Illumina HumanCNV370-Quad v3.0 chips, containing 373,397 probes. The 30 pheochromocytomas/paragangliomas were analyzed with Illumina Human610-Quad v1.0 chips, containing 620,901 probes. Hybridization was performed by IntegraGen (Evry, France), according to the instructions provided by the array manufacturer. Raw fluorescent signals were imported into Illumina BeadStudio software and normalized, as previously described [34], to obtain the log R ratio (LRR) and B allele frequency (BAF) for each SNP. The tQN normalization procedure was then applied to correct for the asymmetry in BAF signals due to the bias between the two dyes used in Illumina assays [35].

### Detection of LOH

Genome-wide copy-number changes in tumors were determined as previously described [12]. The genomic profiles were divided into segments by applying the circular binary segmentation algorithm (DNAcopy package, Bioconductor) [36], [37] to the LRR and BAF signals separately. The allele-specific copy number of each segment was then determined by the Genome Alteration Print (GAP) method [12]. In brief, the GAP pattern of each sample (a sideview projection of segmented LRR and BAF) was constructed, and the best-fitting GAP model was used to determine the ploidy of the sample, the level of contamination with normal cells and the absolute copy number and genotype corresponding to each cluster of segments. We considered a segment to have undergone LOH when the copy number of the minor copy of the segment was <0.5. Note that throughout this paper, LOH refers to any chromosome region in which one of the parental copies has been completely lost, whatever the copy number of the retained chromosome.

### Inferring tumor haplotypes from B allele frequency profiles

In a genomic region of LOH, the BAF profile is either close to 0 (A allele retained) or close to 1 (B allele retained). We used fixed thresholds to reconstruct tumor haplotypes in regions of LOH. SNPs with a BAF>0.65 were assigned the genotype B, whereas SNPs with a BAF<0.35 were assigned the genotype A. We considered SNPs with a BAF between these two thresholds in at least one of the tumors to be unreliable, and removed them from the analysis. We estimated the error rate for haplotype inference by this method, by analyzing previously published data for two tumors from a single patient that displayed LOH for the same chromosomes [13] and comparing the haplotypes reconstructed independently for each sample.

### Detection of significantly recurrent IBD in tumor haplotypes

Once recurrent regions of LOH have been established and tumor haplotypes within these regions have been reconstructed, the FounderTracker pipeline is used to detect haplotypes that are significantly conserved in each candidate region. FounderTracker first assigns an IBD score to each SNP in the region. The significance of each IBD score is then assessed by comparison with a null distribution obtained from a reference set of haplotypes.

#### Detecting pairwise IBD with GERMLINE

The first step in our scoring approach is the identification of pairwise IBD in the set of tumor haplotypes. The GERMLINE algorithm has been shown to be a robust tool for detecting pairwise IBD from phased haplotype data [3]. In this study, we used GERMLINE version 1.5, downloaded from the GERMLINE web page (http://www1.cs.columbia.edu/~gusev/germline/), with default settings, except that: (i) the minimum length for a match (-min_m) was set to 0.4 cM, to allow the detection of short IBD segments, (ii) the number of mismatching markers allowed (-err_hom) was set to 0, because the error rate for the inference of haplotypes from tumor samples was shown to be negligible, (iii) the size of each slice used for exact matching seeds (-bits) was set to 5, to increase the accuracy of IBD boundaries.

#### Calculating the IBD score

The GERMLINE algorithm provides output in the form of a list of pairwise IBD segments with, for each segment, the indices of the first and last probe delineating the segment, and the identifiers of the two samples sharing identical haplotypes. We added a score to each segment to characterize the significance of pairwise IBD. As a series of common alleles is more likely to be identical in two samples by chance, our score takes into account both the length of the segment and the allelic frequencies of the probes defining the common haplotype. Let us denote by *H* a haplotype of *N* SNPs identical in two samples of the dataset. *H* is a vector 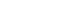 such that *H_i_* represents the allele of the *i^th^* SNP (A or B) in the conserved haplotype. The score *S* associated with the pairwise IBD segment of haplotype *H* is given by the formula below, where *Bfreq_i_* is the allelic frequency of the B allele for the *i^th^* SNP:
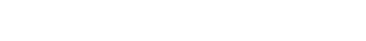



This score corresponds to the log-transformed probability of observing these two identical haplotypes by chance if all SNPs were independent.

An IBD score is then calculated for each SNP *i*, by summing the scores of all the pairwise IBD segments containing that SNP:
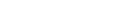



#### Estimating the null distribution of IBD scores

The IBD score described above characterizes the recurrence of IBD at a given SNP location. It takes into account the length of IBD segments and the allelic frequencies of SNPs, but not the linkage disequilibrium between SNPs. This bias is corrected by comparing the IBD score of each SNP with the distribution of IBD scores for the same SNP under the null hypothesis that no haplotype shows significant IBD in the set of samples. This distribution is established by generating 100 reference haplotype subsets, of the same size as the tumor set, by random sampling in a reference data set of phased haplotypes (e.g. from the HapMap [38], [39] or 1,000 Genomes [40] projects). We then calculate the IBD score of each SNP in each of these subsets. The null distribution is then approximated to the Gumbel distribution that best fits the distribution of scores obtained in the 100 reference haplotype subsets. The Gumbel distribution was preferred here to the Gaussian distribution because it yielded a better approximation of the empirical null distributions and more conservative estimates of the p-values. For both the analysis of childhood adrenocortical tumors and pheochromocytomas, we used as reference the 174 phased CEU haplotypes of the 1,000 Genomes project, downloaded together with the recombination rate file from the IMPUTE web page:

https://mathgen.stats.ox.ac.uk/impute/impute_v2.html#download_reference_data.

#### Assessing the significance of IBD scores

In the final step of the pipeline, FounderTracker assigns a p-value to each SNP by comparing the IBD score observed in the tumor set of haplotypes to the null distribution established as described above. This p-value represents the probability of obtaining a higher IBD score by chance than the IBD score obtained for the set of tumor haplotypes. Q-values are calculated from the p-values by the Benjamini-Hochberg method [41], to control for false discovery rate.

### Dash

The DASH (DASH Associates Shared Haplotypes) algorithm [7] builds upon pairwise IBD shared segments to infer clusters of IBD individuals. To detect haplotypes significantly conserved in tumors, we first apply GERMLINE to a combined set comprising the tumor haplotypes together with a set of reference haplotypes (here the CEU haplotypes from the 1,000 Genomes project). We then use DASH to detect clusters of IBD from the pairwise IBD matches identified by GERMLINE. Finally, we test through a Fisher's exact test whether each IBD cluster is significantly more frequent in tumors than in reference haplotypes.

### Assessing the performance of the methods with simulated data

The performance of each method was evaluated by analyzing simulated datasets with IBD segments of different sizes (1, 2 or 5 cM) conserved in various proportions of the samples (2 to 50%). We included, in the simulated data, all the SNPs present on the Illumina 1M-Duov3 beadchip, to model the performance that could be expected with the use of a commercial array containing ∼10^6^ SNPs. For these simulations, we used a set of 548 phased haplotypes from the 1,000 Genomes project, comprising data from the CEU, GBR and TSI panels. This set was split in two sets of 274 haplotypes each, one of which (EUR1) was used as reference data set, and the other (EUR2) was used to generate simulated tumor data sets.

For each simulation, a chromosome was chosen at random, and 100 haplotypes of this chromosome were picked up in the EUR2 haplotype set, to constitute a simulated data set of 100 tumors. A genomic location was then randomly selected on the chromosome, and the haplotype of the first sample for this region was repeated in the desired proportion of samples. In a real data set of tumors harboring a founder mutation, all patients have inherited a different chromosome segment from the founder. These segments all comprise the mutation, but they have different boundaries. To reproduce this variability, the size of the conserved haplotype in each sample was randomly picked up from a Gumbel distribution of location and scale equal to the desired mean haplotype length. Such distribution was found to reproduce well the natural variation in IBD lengths observed around the *TP53* founder mutation in adrenocortical tumors.

FounderTracker and DASH were then applied to the same simulated data, and the rates of true positives (significant SNPs within the conserved haplotype) and false positives (significant SNPs outside the conserved haplotype) were determined at a series of q-value thresholds, with the PresenceAbsence R package [42], to reconstruct the ROC curve associated with each method. For each size and proportion, 100 simulations were carried out and the mean ROC curve was calculated. Power and false discovery rates were calculated using the threshold q = 0.01 for both methods.

### Availability

FounderTracker can be used online through a web application, and the source code can be downloaded from our web page (http://cit2.ligue-cancer.net/FounderTracker). The SNP data from the two tumor datasets analyzed in this study have been deposited in NCBI's Gene Expression Omnibus [43] and are accessible through GEO Series accession number [GEO: GSE32206].

## Supporting Information

Figure S1 **IBD score and significance across the 11p15 region in childhood adrenocortical tumors.** The IBD score in tumors (top) displays some peaks, but these peaks correspond to normal variations due to the pattern of linkage disequilibrium across the region and are also encountered in the null distribution of IBD scores (middle). Thus, no significant IBD is detected in this region (bottom).
(PDF)

Figure S2 **Length of IBD segments between the different pairs of childhood adrenocortical tumors.** This image, exported from the Integrative Genomics Viewer [44], displays the IBD score along the 17p arm (top) and the tumor haplotypes ordered according to the extent of their pairwise IBD with other samples (bottom). The longest pairwise IBD segment, between samples HS_07 and HS10 (green rectangle), spans 8 Mb. By contrast, the smallest pairwise IBD segment, between samples HS_01 and HS_14, spans only 520 kb, defining the minimal haplotype conserved in all samples, corresponding to the peak IBD score.
(PDF)

Figure S3 **Application of FounderTracker (top) and DASH (bottom) to the analysis of chromosome 17 in the childhood adrenocortical tumor dataset.** Both methods detect the conserved haplotype around the *TP53* gene.
(PDF)

Figure S4 **Power analyses of simulated data for the SNP content of Illumina 370K (in blue) or 1M (in red) SNP arrays.**
(PDF)

Figure S5 **Application of FounderTracker (top) and DASH (bottom) to the detection of significantly recurrent IBD on the 11q arm of 11 pheochomocytomas and paragangliomas.**
(PDF)

Table S1 Clinical data for the 30 pheochromocytoma and paraganglioma samples.
(XLS)
