# Supplementary figures and images for: Identity by Descent Mapping of Founder Mutations in Cancer Using High-Resolution Tumor SNP Data
Source: PLoS One. 2012 May 2;7(5):e35897. doi: 10.1371/journal.pone.0035897 (PMC3342326; doi:10.1371/journal.pone.0035897)

# 11p15 region

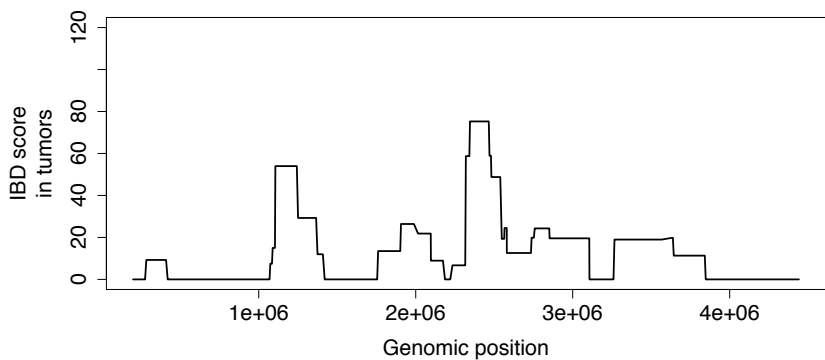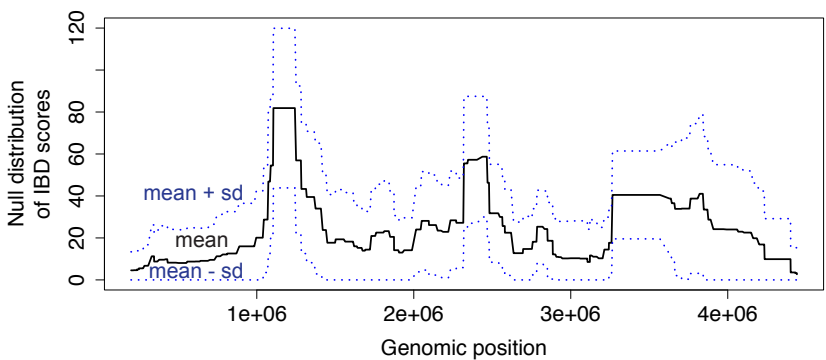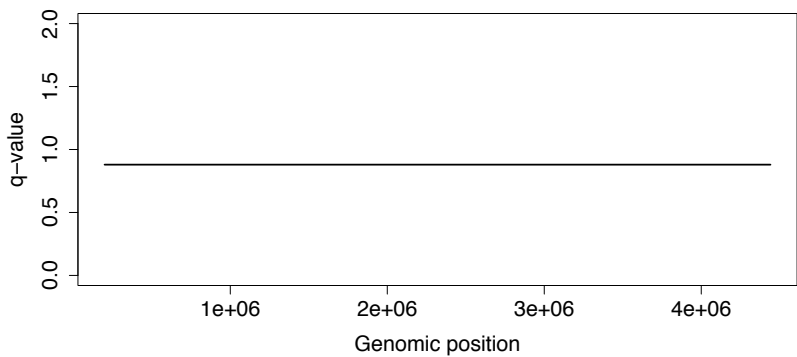

Supplement: Figure S1 — IBD score and significance across the 11p15 region in childhood adrenocortical tumors. The IBD score in tumors (top) displays some peaks, but these peaks correspond to normal variations due to the pattern of linkage disequilibrium across the region and are also encountered in the null distribution of IBD scores (middle). Thus, no significant IBD is detected in this region (bottom). (PDF) [file pone.0035897.s001.pdf]

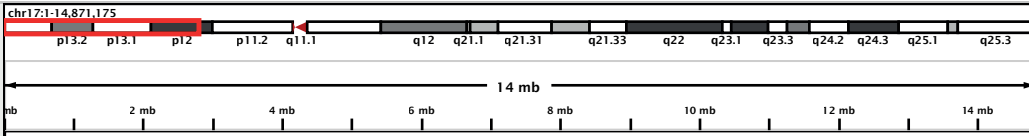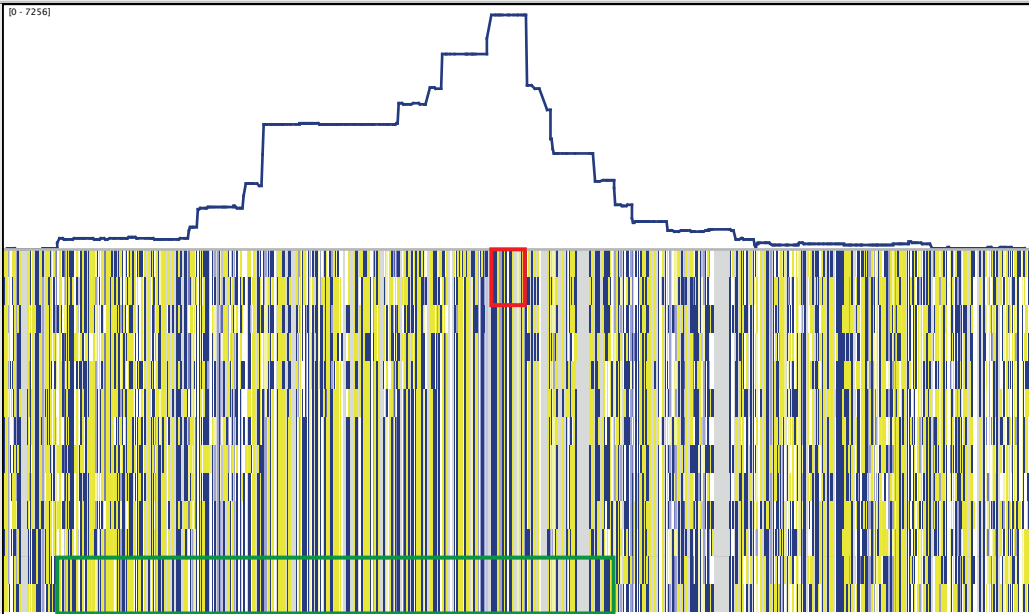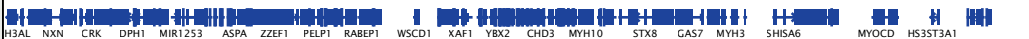

Supplement: Figure S2 — Length of IBD segments between the different pairs of childhood adrenocortical tumors. This image, exported from the Integrative Genomics Viewer [44], displays the IBD score along the 17p arm (top) and the tumor haplotypes ordered according to the extent of their pairwise IBD with other samples (bottom). The longest pairwise IBD segment, between samples HS_07 and HS10 (green rectangle), spans 8 Mb. By contrast, the smallest pairwise IBD segment, between samples HS_01 and HS_14, spans only 520 kb, defining the minimal haplotype conserved in all samples, corresponding to the peak IBD score. (PDF) [file pone.0035897.s002.pdf]

# Chromosome 17

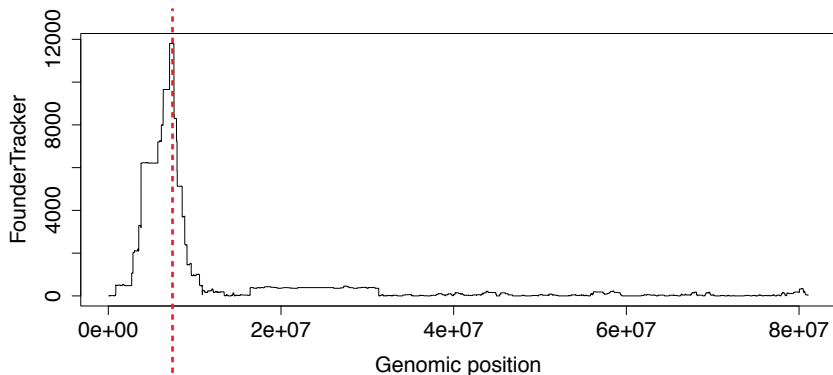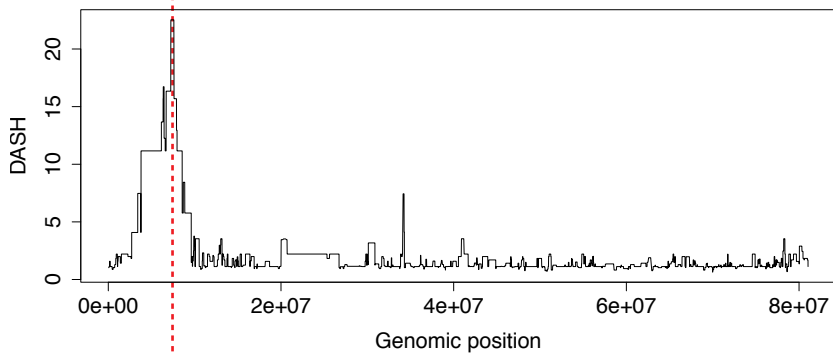

*TP53*

Supplement: Figure S3 — Application of FounderTracker (top) and DASH (bottom) to the analysis of chromosome 17 in the childhood adrenocortical tumor dataset. Both methods detect the conserved haplotype around the TP53 gene. (PDF) [file pone.0035897.s003.pdf]

**0.5 cM**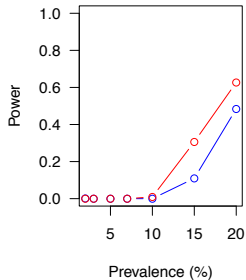**1 cM**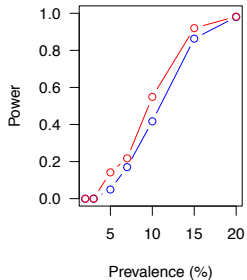**2 cM**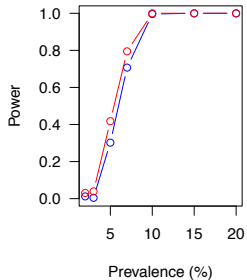**5 cM**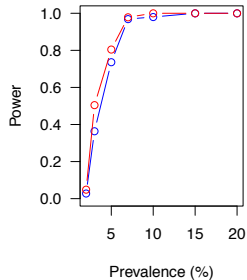

— Illumina CNV370 (373,397 markers)

— Illumina 1M (1,199,187 markers)

Supplement: Figure S4 — Power analyses of simulated data for the SNP content of Illumina 370K (in blue) or 1M (in red) SNP arrays. (PDF) [file pone.0035897.s004.pdf]

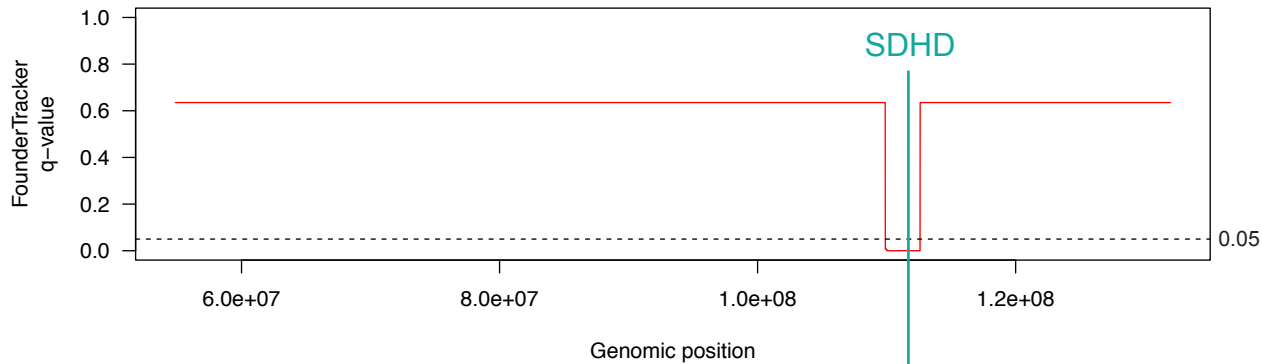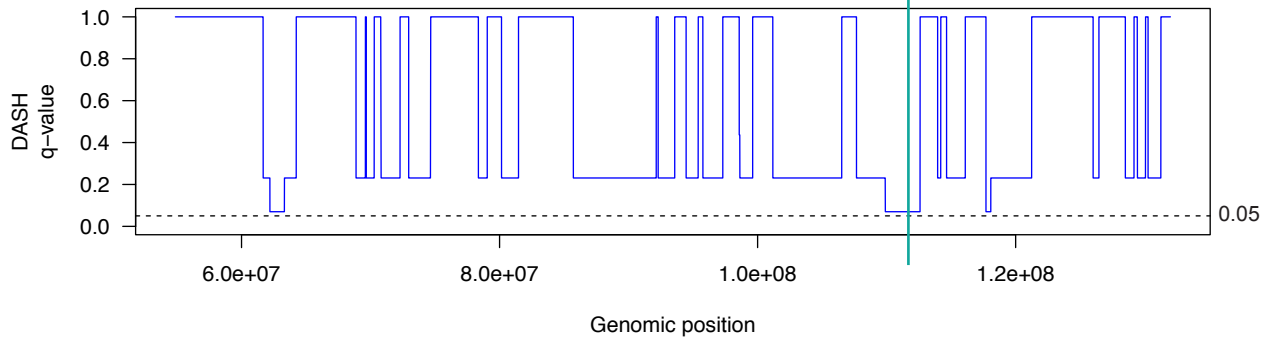

Supplement: Figure S5 — Application of FounderTracker (top) and DASH (bottom) to the detection of significantly recurrent IBD on the 11q arm of 11 pheochomocytomas and paragangliomas. (PDF) [file pone.0035897.s005.pdf]
